# Seismic imaging of a basaltic Lesser Antilles slab from ancient tectonics

**DOI:** 10.1038/s41586-025-08754-0

**Published:** 2025-04-09

**Authors:** Xusong Yang, Yujiang Xie, Catherine A. Rychert, Nicholas Harmon, Saskia Goes, Andreas Rietbrock, Lloyd Lynch, Members of the VoiLA Working Group, Colin G. Macpherson, Jeroen Van Hunen, Jon Davidson, Marjorie Wilson, Robert Allen, Jenny Collier, Jamie J. Wilkinson, Timothy J. Henstock, John-Michael Kendall, Jonathan D. Blundy, Joan Latchman, Richard Robertson

**Affiliations:** 1 State Key Laboratory of Lithospheric and Environmental Coevolution, Institute of Geology and Geophysics, Chinese Academy of Sciences, Beijing, China; 2 College of Earth and Planetary Sciences, University of Chinese Academy of Sciences, Beijing, China; 3 Ocean and Earth Science, University of Southampton, Southampton, UK; 4 Rosenstiel School of Marine, Atmospheric, and Earth Science, University of Miami, Miami, FL USA; 5 Institute for Geophysics, Jackson School of Geosciences, The University of Texas at Austin, Austin, TX USA; 6 Woods Hole Oceanographic Institution, Falmouth, MA USA; 7 Department of Earth Science and Engineering, Imperial College London, London, UK; 8 Karlsruhe Institute of Technology, Karlsruhe, Germany; 9 Seismic Research Centre, The University of the West Indies, St. Augustine, Trinidad and Tobago; 10 Department of Earth Sciences, Durham University, Durham, UK; 11 Institute of Geophysics and Tectonics, School of Earth and Environment, University of Leeds, Leeds, UK; 12 Department of Earth Sciences, University of Oxford, Oxford, UK

**Keywords:** Seismology, Geophysics

## Abstract

At subduction zones, lithospheric material descends through the upper mantle to the mantle transition zone (MTZ), where it may continue to sink into the lower mantle or stagnate^1,2^. Several factors may be important in influencing this flow, including chemical heterogeneity^3–5^. However, tight constraints on these mantle flows and the exact factors that affect them have proved challenging. We use P-to-S receiver functions to image the subducting slab and the MTZ beneath the Lesser Antilles subduction zone. We image a singular, superdeep (>700 km) 660-km discontinuity over a 200-km-wide zone within the slab, accompanied by nearby double 660 discontinuity phases (normal and superdeep). Combined geodynamic and waveform modelling shows that this observation cannot be explained by temperature effects in typical mantle compositions but requires a large basalt-rich chemical anomaly, strongest in the location of the singular, deep 660. The inferred basalt signature is near the proposed location of a subducted extinct spreading ridge^6,7^, where basalt is probably present in greater proportions. Our finding suggests that past tectonic events impart chemical heterogeneity into slabs, and the heterogeneities, in turn, may affect the inherent tendency of the slab to sink.

## Abstract (web summary)

Imaging of the mantle transition zone beneath the Lesser Antilles shows a basalt-rich region within the subducting slab near the proposed location of a subducted extinct spreading ridge, implying ancient tectonics play a role in influencing slab trajectories.

## Main

The MTZ is the region located between 410 km and 660 km depth and bounded by two prominent global seismic velocity discontinuities, herein referred to as ‘the 410’ and ‘the 660’, respectively^8^. The discontinuities are typically thought to represent solid-state phase transitions in dominant mantle minerals: from olivine to wadsleyite (Wd) at approximately 410 km depth and from ringwoodite (Rw) to perovskite at approximately 660 km depth^9^. The MTZ is predicted to be thinner in hotter regions, for example, below hotspots, and thicker in colder regions, for example, near subduction zones, owing to the pressure–temperature dependence of these phase transitions^10^. Several seismic observations support this classic model of higher temperatures below hotspots owing to upwellings from the lower mantle to the upper mantle and colder temperatures in areas of recent or continuing subduction owing to slab downwellings from the upper mantle to the lower mantle^11,12^.

Although there is agreement that the mantle is composed of at least 50–60% olivine or its polymorphs, with an average mantle composition close to that of pyrolite, it is debated to what extent and on what scales mantle composition is heterogeneous^3^. Different studies propose that the mantle largely consists of either an equilibrium pyrolitic composition or an on-average pyrolitic mixture of basalt and harzburgite^4,13^, or laterally varying degrees of equilibrium assemblages and mechanical mixtures^14,15^. The distribution of compositional heterogeneity has important implications for our understanding of the relationship between mantle conditions, large-scale mantle convection patterns, and the cycling of chemical components through Earth’s interior^3^.

Subducting slabs play a key role in mantle flow^16^ and insert compositional heterogeneity into the mantle^17,18^. Subducting slabs consist of a magmatic oceanic crustal layer overlying a melt-depleted mantle lithosphere. The mantle lithosphere may also be heterogeneous. For instance, frozen melt sills are seismically imaged within both young and old lithosphere, for example, within the Juan de Fuca Plate^19^ and the Pacific Plate^20^. Also, melt channels at the lithosphere–asthenosphere boundary have been inferred on the basis of seismic and magnetotelluric imaging beneath both young and old seafloor^21,22^. Particularly at slow-spreading ridges such as that in the Atlantic, magma supply and accumulation may be variable in time^23,24^. Yet, we have few high-resolution images from subduction zones consuming lithosphere formed by slow spreading, as there are only two Atlantic subduction zones and large-scale ocean-bottom deployments are required.

**Fig. 1:**
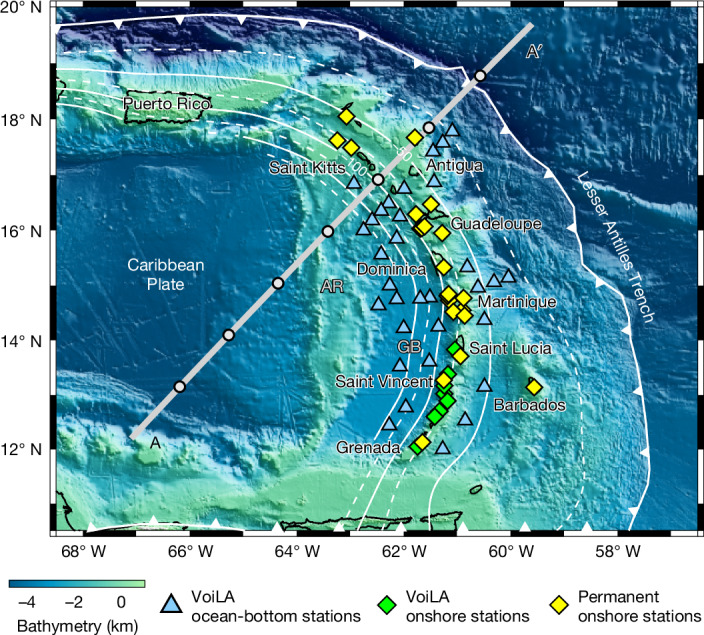
Tectonic setting and seismic networks used in this study. Background colours illustrate bathymetry. The thin white contours (dashed and solid) depict slab geometry from the SLAB2.0 model^46^. Line with triangles indicates the position of the trench. The grey line across the map shows the location of the vertical cross-section shown in Fig. 3. The dots along the grey line represent the central locations of the bins for the stacked receiver functions shown in Fig. 3. Seismic stations are depicted by the diamonds and triangles. AR, Aves Ridge; GB, Grenada Basin.

We use P-to-S wave receiver functions to image the MTZ discontinuities of the Lesser Antilles subduction zone in the western Atlantic using ocean-bottom seismic data collected as part of the Volatile Recycling in the Lesser Antilles (VoiLA) project^25^ and other available land data (Fig. 1). At this subduction zone, lithosphere formed at the slow spreading Mid-Atlantic and now almost fully subducted Proto-Caribbean Ridges has been subducting for roughly the past 120 Myr (ref. ^6^).

**Fig. 2:**
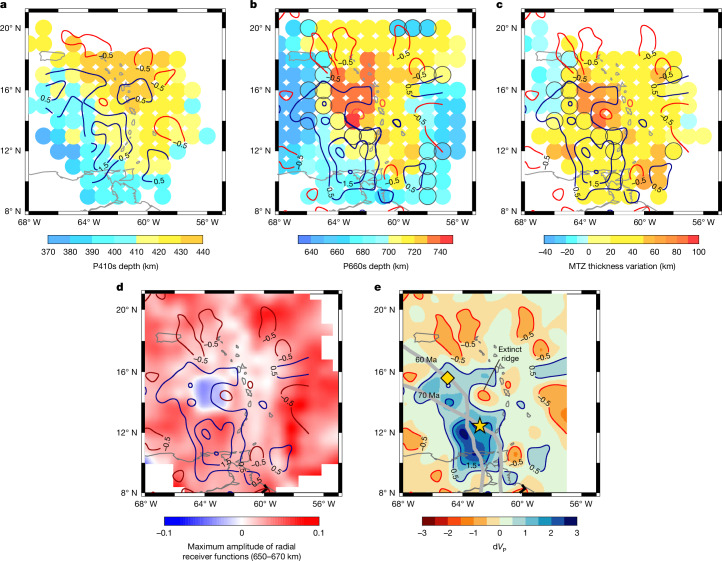
Map view of binned depths and amplitudes of transition zone seismic phases. Dots in panels **a**–**c** indicate the bin locations. The coloured contours in all panels represent the P-wave anomalies of model VoiLA-P19 at respective depths^6^. **a**, Depth of the 410 from P410s. **b**, Depth of the 660 from P660s. **c**, MTZ thickness. In panels **b** and **c**, dots with a black ring denote the presence of double phases around 660 km depth (as illustrated in Extended Data Fig. 1). In panels **b** and **c** for the bins where double discontinuities are imaged, the shallower case is shown and otherwise the singular super deep phase depth is shown, whereas results based on the deeper phase in the double discontinuity bins are shown in Extended Data Fig. 2. Panel **d** shows the maximum amplitude of the stacked radial receiver functions between 650 km and 670 km, with the blue area highlighting where the DNP exists, that is, the 660 ‘gap’. The grey curves in panel **e** show reconstructed positions of the Proto-Caribbean slab subducted 60 and 70 Ma (ref. ^6^). The yellow diamond shows the location of the extinct spreading ridge, and the yellow star indicates where the spreading ridge subducted while still active, forming a slab window. Source data

**Extended Data Fig. 1:**
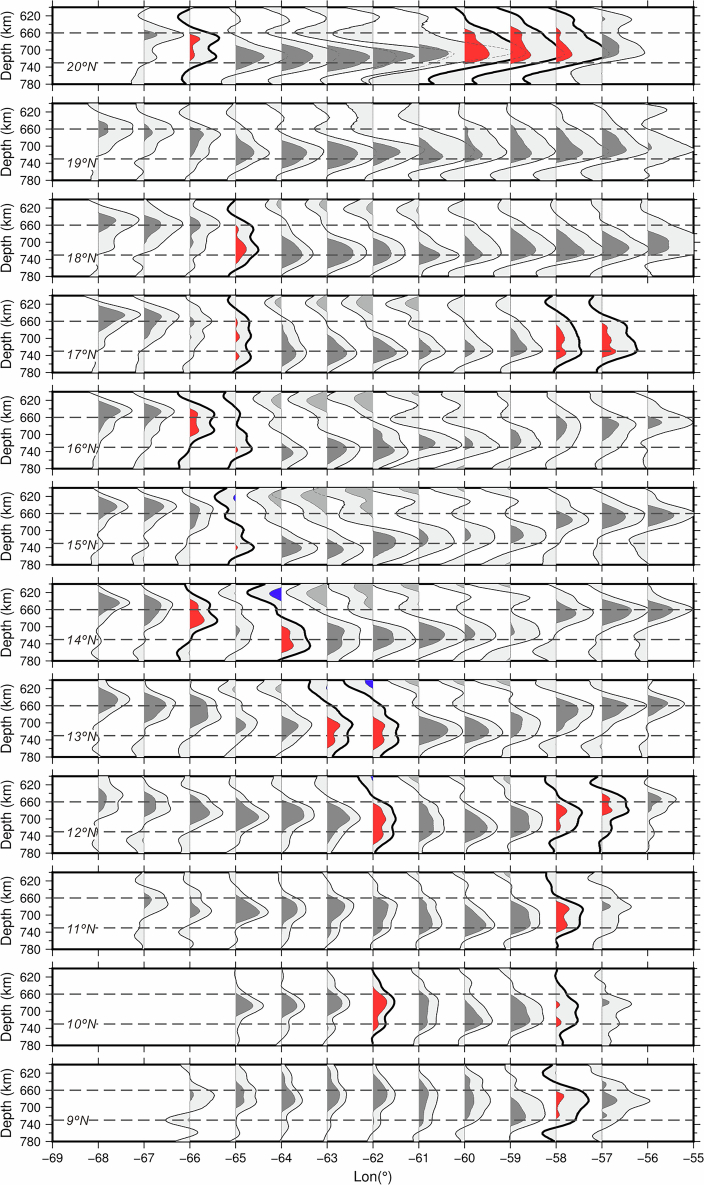
Stacked receiver functions for each bin. Stacked receiver functions for each bin presented in Fig. 2b. The error bars correspond to the 95% confidence region of the stacked waveforms in each bin. Receiver functions with bold lines and red shading indicate the presence of double 660s. Source data

**Extended Data Fig. 2:**
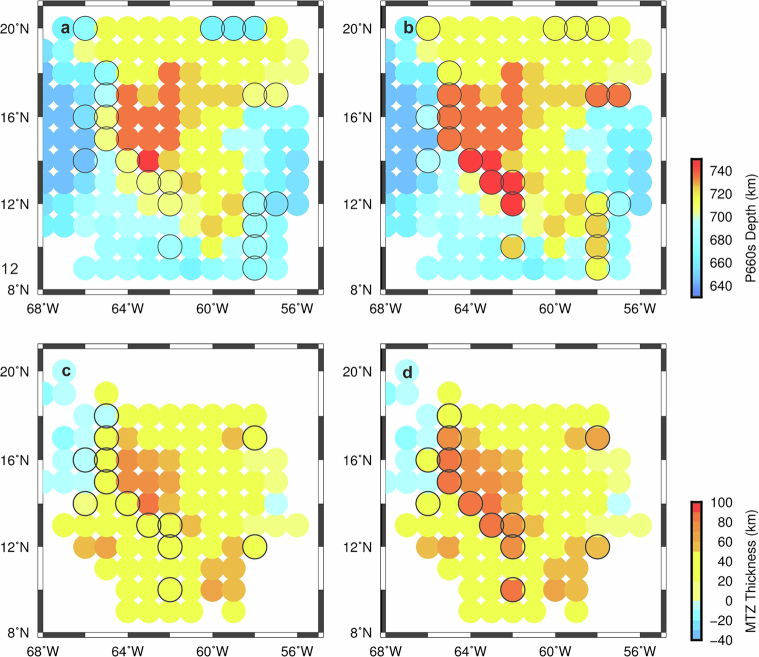
Map view of the 660 discontinuity topography and the thickness of the MTZ. Dots with a black ring indicate the presence of double phases around 660 km depth. Panels **a** and **c** show the 660 discontinuity topography and the MTZ thickness based on the upper phase of the double 660, respectively. Similarly, panels **b** and **d** illustrate the 660 discontinuity topography and the MTZ thickness based on the lower phase of the double 660, respectively. Bins characterized by double 660 discontinuities, one at a more normal depth and a superdeep phase, are outlined in black. Source data

We image positive peaks (velocity increases with depth) associated with the 410 and 660 discontinuities across a broad area below the Lesser Antilles. We find that the 410 is uplifted by 10–30 km in an area with an approximate NNW strike beneath the southwestern Lesser Antilles and depressed (to 410–440 km depth) in the northeast, with the strongest depression occurring in the region below the northern Lesser Antilles Islands (Fig. 2a). The 660 phases are mostly depressed (to 660–743 km depth) across the study region, with some exceptions near the western and eastern edges (Fig. 2b). We divide the depressed 660 into two categories: the deep 660 (depth between 660 km and 700 km) and the superdeep 660 (depth greater than 700 km). A superdeep 660 occurs over a 400-km-wide swath below the backarc and the northern part of the forearc region. In a 200-km-wide circular region in the backarc behind Dominica and Martinique (Fig. 2b), only a singular superdeep phase (depth greater than 700 km) is observed, resulting in an unusually thick MTZ there (>300 km) (Fig. 2c). However, in some of the bins with superdeep 660 phases, there are also shallower, more normal 660 phases, leading to double 660 discontinuities (in the waveforms in Extended Data Fig. 1, in map view as bins circled in black in Fig. 2b, and Extended Data Fig. 2). Double 660 bins occur consistently on the western side of the singular, superdeep 660 region and sporadically to the east.

Deep negative phases (velocity decreases with depth), herein referred to as ‘DNPs’, are observed within the MTZ at 588–622 km depth in the areas with superdeep 660 discontinuities. The phases result in an apparent polarity reversal in the receiver function model from positive to negative in this depth range (Fig. 2d). This region also coincides with a slow-wave-speed gap in the fast slab anomaly imaged by body-wave tomography (Fig. 2e, circular, red −0.5% body-wave tomography anomaly contour that is surrounded by blue slab contours) and is near the estimated location of a subducted ridge segment, at which spreading is thought to have stopped about 70 million years ago (Ma)^6,7,26^ (Fig. 2e, yellow diamond and star).

**Extended Data Fig. 3:**
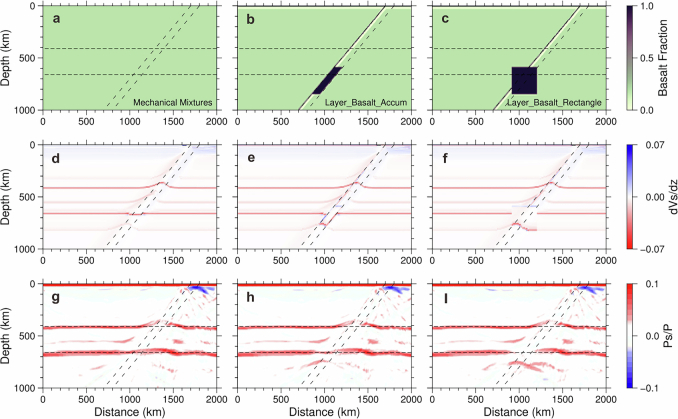
Full-waveform seismic tests. Panels **a**–**c** illustrate the compositional fields of cases MM, LBA and LBR, respectively. Corresponding shear wave velocity gradient fields are shown in panels **d**–**f**, whereas the migrated synthetic receiver function results are presented in panels **g**–**i**. A superdeep 660 only appears in models with a large basalt accumulation in the slab.

Resolution testing shows that our interpreted results are robust (Methods). We note that the presented transect is located in the section of the arc with less curvature between 14° N and 18° N (Fig. 1). We also tested possible effects of assumed migration models to show that the imaged lateral depth variabilities of the interfaces are unlikely to be an artefact of assumed velocities. However, observed MTZ thicknesses are also greatest in the locations of the superdeep 660s (Fig. 2), showing that the result is not an artefact of upper-mantle velocities assumed in the migration model. Similarly, MTZ thickness is consistent with 2D full-waveform modelling of the P-SV system that shows that the observed superdeep 660 phases and DNPs and their apparent spatial extents are not artefacts related to the interaction of the wavefield with the slab (Extended Data Fig. 3).

**Fig. 3:**
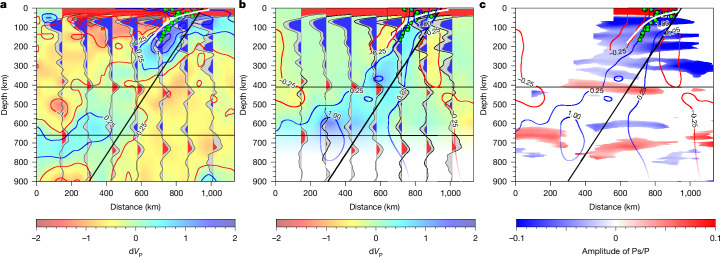
Vertical cross-section through P-wave velocity models and the migrated receiver functions. **a**, Vertical cross-section showing the P-wave velocity anomalies of model UU-P07 (ref. ^47^) overlain by depth-migrated receiver functions (black wiggles) from binned areas shown in Fig. 1. Red (positive), blue (negative) and shaded parts (95% confidence levels) of the receiver function are shown. **b**, Vertical cross-section showing the P-wave anomalies of model VoiLA-P19 (ref. ^6^) overlain by receiver functions. **c**, Vertical cross-section through the migrated receiver functions. Grids with confidence level <95% are masked to emphasize robust phases. Results for the grid without this cutoff are presented in Extended Data Fig. 7. The white line delineates the top of the descending slab according to the SLAB2.0 model^46^. Seismicity from the relocated VoiLA dataset^48^ is shown as green dots. The black line roughly delineates the location of the slab in body-wave tomography (**a**,**b**). Source data

The 410-km discontinuity is attributed to the phase transition from olivine to Wd and its depth variation is probably the result of variations in temperature^10^ and/or water content^27^. The uplifted 410s near the subducted Proto-Caribbean slab coincide with the high-velocity slab-like anomalies in the VoiLA-P19 model^6^ (Fig. 2a) and, therefore, can probably be explained by expected cold slab temperatures (Methods). Behind the slab, our depressed 410 observations correspond to regions of low-velocity anomalies^6,28^ (Figs. 2a and 3a,b). This could be related increased hydration and/or hotter temperatures owing to upwelling (Methods), although this is not an integral part of our conclusions.

The lack of any anomaly in the depth of the 660 in the east and the slight uplift in the west (Fig. 2b) may be explained by a combination of factors (Methods), although again neither is particularly important to our conclusions.

**Extended Data Fig. 4:**
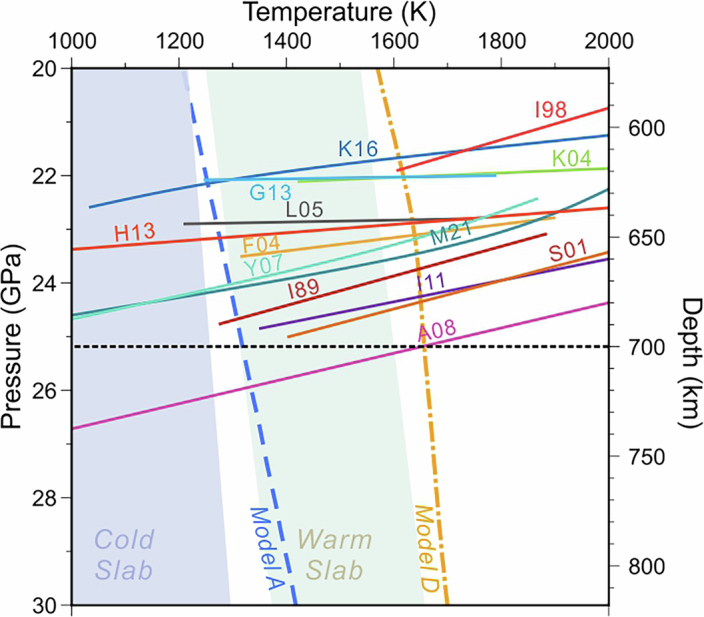
Phase boundaries for the post-spinel transition (ringwoodite to bridgmanite and periclase). Modified after ref. ^31^. I89 (ref. ^10^), I98 (ref. ^71^), S01 (ref. ^72^), K04 (ref. ^73^), F04 (ref. ^74^), L05 (ref. ^75^), Y07 (ref. ^76^), A08 (ref. ^77^), I11 (ref. ^78^), G13 (ref. ^79^), H13 (ref. ^80^), K16 (ref. ^81^), I18 (ref. ^29^), M21 (ref. ^31^).

**Extended Data Fig. 5:**
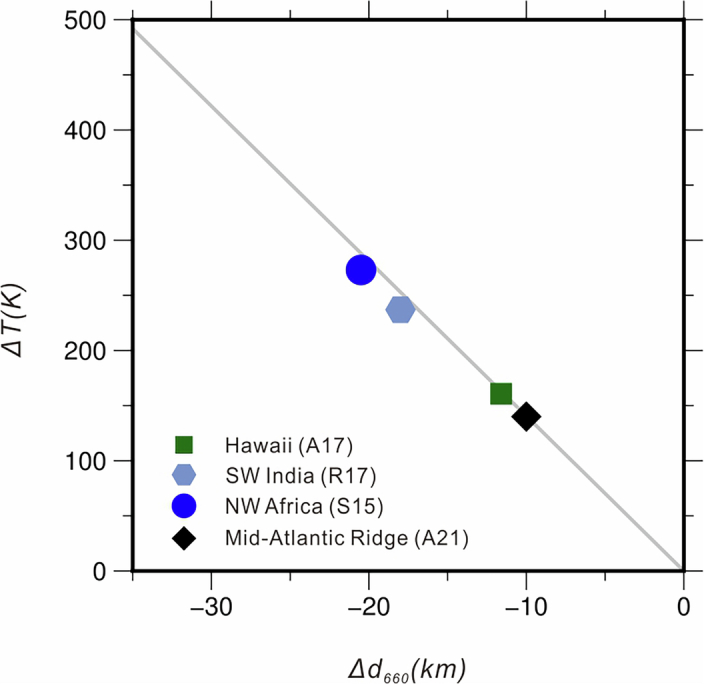
Temperature estimates from relationships with the 660-discontinuity topography. Grey line is the Clapeyron slope of −2.5 MPa M^−1^ (ref. ^82^). Symbols represent average estimates from the Mid-Atlantic Ridge^83^, northwest Africa^84^, Southwest Indian Ridge^85^ and Hawaii^86^.

Seismologically observed depressions of the 660 in subduction zones are usually attributed to thermal effects on the dissociation of Rw (ref. ^29^). This could explain the 10–20-km depression of the 660 in the southwest of the Lesser Antilles, given the good spatial agreement with the location of high-velocity anomalies in the tomography models (Fig. 2). The depression of the 660 (approximately 20 km) is also in agreement with previous imaging of the MTZ discontinuities in the southwest^30^. However, the dissociation of Rw is expected to occur at <700 km depth for the range of thermal predictions of the Antilles slab for transition zone depths (Extended Data Figs. 4 and 5). Therefore, the superdeep 660 cannot result purely from thermal effects.

**Extended Data Fig. 6:**
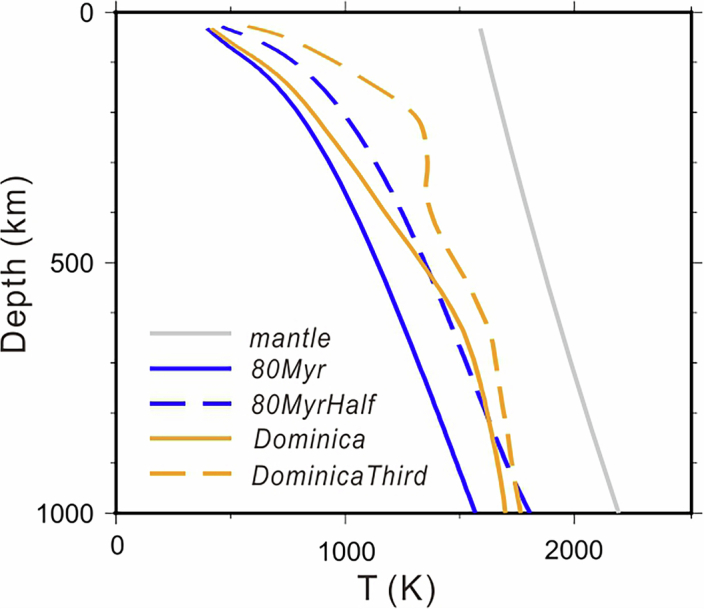
Slab geotherms of different models. Geotherms follow the minimum in slab temperatures. Model A, 80 Myr: constant subducting-plate age at the trench of 80 Myr, constant convergence velocity of 2 cm per year. Model B, 80 MyrHalf: as model A but with halved slab-sinking velocities. Model C, Dominica: variable age and velocity at trench representative of subduction near Dominica. Model D, DominicaThird: as model C but with one-third of the sinking velocity of the slab. For comparison, the mantle adiabat is shown in grey.

Other factors can affect the depth of the Rw transition, including water content^31^ and/or very sluggish kinetics leading to the persistence of metastable Rw (ref. ^32^). Increased water content may steepen the Clapeyron slope of the Rw transition and/or depress the depth of the 660 (ref. ^29^). However, previous studies^31^ predict this effect to be mild (<8 km) and hence it is unlikely to be sufficient to generate the superdeep 660. The persistence of metastable Rw into the perovskite stability fields may depress the 660 discontinuities by 20–25 km at very low MTZ temperatures^32^, around 975 K. Geodynamic modelling using region-specific subducting ages and convergence rates (Methods) suggests that the coldest core of the slab is 1,290–1,660 K at 660–700-km depth, that is, far from the required 975 K (Extended Data Fig 6; Methods). Therefore, the Proto-Caribbean slab is highly unlikely to be cold enough for the superdeep 660 to be the result of metastable Rw.

**Fig. 4:**
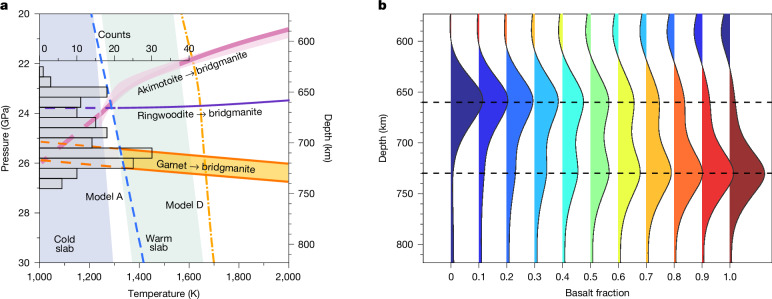
Phase transition boundaries and seismic modelling results. **a**, Predicted phase transitions near 660 km depth. Phase boundaries for the Rw transition and the Ak transitions are from ref. ^34^. Phase boundaries for the Gt transition in mid-ocean ridge basalt (MORB) composition are from ref. ^33^. Note that the Gt transition is only expected to be strong at high basalt fractions. The histogram of the 660 depths from this study is shown by grey bars. **b**, Predicted synthetic receiver functions with different basalt fractions along an Antilles slab geotherm (see Methods).

The non-olivine, or, in other words, the ‘garnet-pyroxene’ components, of mantle compositions are also predicted to undergo phase transitions near 700 km depth, including the akimotoite (MgSi-ilmenite) transition to perovskite^33–35^ and the garnet (Gt) transition to perovskite^33,35^. The akimotoite (Ak) transition cannot explain the observed superdeep 660 here because very cold temperatures, <1,100 K, are required to move the Ak-related boundary deeper than 700 km (ref. ^34^), far below the temperature predicted beneath the Lesser Antilles (Fig. 4a).

Alternatively, if the Gt transition would be dominant it could explain the observed superdeep 660 (Fig. 4a). For typical olivine-polymorph-dominated compositions, the Gt transition is dominant at very high temperature conditions^29^, about 2,100 K. However, the Gt transition could be prominent for the thermal conditions predicted for our study area if there is a high concentration of eclogitized basalt.

To quantify the amount of basalt that would be needed to explain our result, we performed testing using synthetic receiver functions. We created velocity models for the minimum and maximum predicted Antilles slab temperatures and various basalt fractions between 600 km and 800 km depth and a pyrolitic mechanical mixture at all other depths (Methods). The Rw-related discontinuity occurs at about 660 km and weakens with increasing basaltic content as the deeper Gt-related phase at roughly 730 km depth begins to dominate (Fig. 4b). Therefore, the observed double 660 observations (bins circled in black in Fig. 2b) are probably explained by moderate basalt fractions, whereas singular superdeep 660 observations (>730 km depth; Fig. 2b) require a basalt anomaly of >50% (Fig. 4b). A negative phase is also predicted to occur at the top of a low-velocity basalt-rich layer, in this example at 600 km depth, albeit with a smaller amplitude than the observed DNP waveform. We are not interpreting absolute amplitudes here. However, oxidation or a lower Ca content of Gt, would further lower the Vs of the basalt-rich layer from 2.5–3.5% to 3.5–7.6% below that of the peridotite mantle^36^, producing a larger DNP prediction.

Our required basalt fractions at 660 km depth are higher than the global average of about 30–40%, as suggested by seismic analyses^4,37^ and geodynamic modelling^5^; however, there are plausible explanations for such high concentrations in this region. Several previous seismic studies have proposed that enriched basalt reservoirs may exist in the vicinity of subduction zones^4,15,38^. For instance, basalt concentrations have been inferred to be as large as 50–80% in the general Caribbean region^15^. Such an abundance is usually assumed to be the result of basaltic crust peeling off the top of the plate^39^. However, geodynamic modelling does not support an easy separation of the basaltic crust at MTZ depths^5,40,41^, fuelling the continuing debate about the origin of chemical heterogeneities within the MTZ. Notably, our DNP phases and superdeep 660 phases are not located in front of the slab anomaly imaged by body waves. Instead, they seem to be within it and partly behind it (Fig. 3). Therefore, what is new about our result is that the inferred basalt accumulation is more closely related to compositional heterogeneity within the slab caused by its tectonic history rather than peeled-off crustal material in front of it.

In particular, the inferred basalt concentration may be related to the nearby inferred subducted extinct ridge. Sub-ridge lithosphere is characterized by relatively high basalt fractions given that the underlying mantle lithosphere is thinner than that beneath older aged lithosphere. Also, further melts may have preferentially frozen into the lithosphere^23^, unable to escape when the spreading stopped 70 Ma (ref. ^6^), further increasing the basalt content in this region. Therefore, basalt-enriched anomalies within the slab, probably originating from ancient tectonic processes near the extinct spreading ridge, may introduce substantial chemical heterogeneities into the MTZ.

Previous studies have proposed a range of slab–MTZ interaction scenarios for this region. Slab rollback has been proposed on the basis of observations from regional seismic, magnetic and geodetic datasets^42–44^, albeit in varying degrees, and geodynamic models typically predict slab stagnation in the MTZ in subduction zones in which rollback occurs^2^. Alternatively, a recent seismic tomography study suggests that rollback is relatively minimal and that substantial slab buckling occurs in the MTZ in this region^6^. Plate buckling could enhance basalt content, which would be consistent with our observations. Basalt may also play a role in enhancing plate stagnation and/or buckling in the vicinity of the extinct ridge, given its predicted lower density compared with the ambient mantle at about 660–750 km depth^45^. This, in turn, may explain the observation of enhanced basalt over a roughly 600-km-wide zone, exceeding that expected for an approximately 200-km slab descending directly into the lower mantle.

Overall, our results indicate the presence of high basalt fractions within the MTZ beneath the Lesser Antilles, which are unlikely to result from delaminated basaltic crust but could instead be associated with the slab itself, potentially related to the extinct ridge and/or the freezing of melts within the lithosphere. This finding suggests that compositional heterogeneities within the slab play a role in dictating the degree to which slabs stagnate in the MTZ or pass through to the lower mantle.

## Methods

### Seismic data

**Extended Data Fig. 8:**
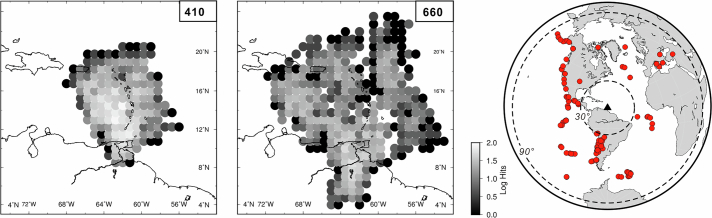
Ps receiver function hit count map and the earthquake sources (red dots) used in this study. In the left two frames, grey shading indicates the number of waveforms averaged into the bins in log_10_(hits). Right panel shows earthquake locations used between 30° and 90° from our study region.

The seismic data used in this study are from two sources: (1) eight broadband stations and 34 ocean-bottom seismometers (OBSs) deployed as part of the VoiLA experiment^25^ from 2016 to 2017 and (2) 22 permanent stations on the Lesser Antilles islands with data publicly available through the Incorporated Research Institutions for Seismology (IRIS) Data Management Center (DMC) (Fig. 1). Waveforms of teleseismic events with magnitude greater than 5.4 M_b_ and with epicentral distances of 30–90° were used to calculate receiver functions (Extended Data Fig. 8). The ocean data were corrected for tilt and compliance noise^49,50^. Original horizontal components and vertical components waveforms were rotated into the P and S components using a transform matrix^51^. For rotation of the land stations, we assumed that the near-surface velocity of the P wave is equal to 5.5 km s^−1^ and that of the S wave is equal to 3.2 km s^−1^. For the rotation of the ocean stations, we used a sediment velocity model based on the P-to-S delay times from the sediment conversion^52^. For the stations without sediment-velocity constraints, the sediment velocities were deduced from the published sediment thickness^52^ by means of velocity–thickness relationships^53,54^. We assumed that the density of sediments is 2,900 kg m^−3^. All waveforms were filtered by applying a zero-phase, fourth-order Butterworth filter from 0.05 to 0.20 Hz. Other processes included removing the mean, removing the linear trend and applying a symmetric taper to each end of data. Then the pre-processed waveforms were manually inspected to retain records with a clear P-wave phase in the P component within 5 s of the theoretical arrival.

### Receiver functions

P-to-S (Ps) receiver functions were determined to reveal the MTZ discontinuities and thickness beneath the Lesser Antilles. An iterative, time-domain deconvolution method with Gaussian width of 0.7 s was applied in the calculation of the receiver functions^55^. Naturally, data from island stations tend to be noisier than those of stations from a stable continent, and this is especially true for OBS stations, thus careful selection was required. Each radial receiver function was inspected manually and only waveforms with a clear Ps phase amplitude for 410 and 660 discontinuities (P410s and P660s, respectively) were selected, which were determined by considering two factors: (1) whether the arrival time on the seismograms is close to the theoretical arrival and (2) whether the arrival time is comparable with that in the neighbouring seismograms. To maximize the use of as many waveforms as possible, waveforms with one clear 410 or 660 phase were selected for the separate datasets of P410s and P660s. In total, 62 stations provided 2,759 and 2,855 waveforms of 143 teleseismic earthquakes for the P410s dataset and the P660s dataset, respectively. The hit map from the respective datasets is shown in Extended Data Fig. 8.

### Depth migration

The receiver function waveforms were migrated to depth and stacked on a 0.75° × 0.75° grid, with a depth spacing of 1 km. For migration, we used a crust-corrected, 3D regional reference model VoiLA-P19 (ref. ^6^). We extended the area outside the 3D model with the 1D Earth reference model AK135 (ref. ^56^). For the ocean stations, we used the sediment thickness and *V*_S_ model that we used for the rotation. We used an average ocean crustal thickness (7 km) with typical velocities (*V*_P_ = 6.82, *V*_S_ = 3.96). For the land stations, the crustal velocity models were obtained from a previous study^57^. The station elevations were corrected relative to sea level. The migrated receiver functions were back-projected along the theoretical ray path and stacked onto a 3D grid. Only grids with more than five waveforms were used. Then the grid was smoothed over the Fresnel zone of the waves with a minimum Fresnel zone cutoff of 50 km. Because the P410s and P660s phases were selected separately, we generated two grids and then merged them by linear weighting from 410 km to 660 km. The study area was uniformly divided into circular bins that overlap and have a radius of 2° and a 1° spacing between them. Receiver functions were stacked within these bins to define the depths of the 410 and 660 interfaces within the study area (Fig. 2). The 410 and 660 depths are not particularly well correlated, with a correlation coefficient of 0.49, indicating the effectiveness of the 3D model used in the migration process. We also calculated the MTZ thickness within each bin, with uncertainties substantially less than in depths of the individual discontinuities (Fig. 2). The results show that the deep 660 anomalies we interpret coincide with areas of particularly thick MTZ. This implies that the deep 660 anomalies are unlikely to be artefacts induced by uncertainties in the migration process. Similarly, focusing from curvature does not likely dominate our results. Migration in 3D mitigates this to some degree. Moreover, our transects are focused on a relatively straight section of the Lesser Antilles. Also, there is no reason to believe that this would necessarily result in the spatial agreement of our anomalies with those of body-wave tomography and the extinct ridge.

We tested the degree of agreement between the ocean stations and land stations and the effect of different migration models. We tested using the AK135 model^56^, with a modified crust. The features we interpreted are in good agreement with the results of different models and different datasets, indicating that they are robust. We also tested using the VoiLA model and AK135 model and a range of *V*_P_/*V*_S_, that is, increase from 0.10 to 0.05 compared with AK135 *V*_P_/*V*_S_. The features interpreted here are present in all of these tests, which suggests that they are robust features. The depths of both P410s and P660s phases get shallower with the increase of *V*_P_/*V*_S_ and the trend is basically linear. The standard deviation (*σ*) for each grid point is used to determine the confidence level. We interpreted phases with a confidence level exceeding 95% (>2*σ*).

### Uplifted 410 near the slab and depressed 410 behind the slab

Behind the slab, our depressed 410 observations correspond to regions of low-velocity anomalies^6,28^ (Figs. 2a and 3a,b). This could be explained by either increased hydration or hotter temperatures owing to upwelling. For instance, hydration could cause seismically slow velocities, although, upwellings may be required to explain the depressed 410. Typically, geodynamic models predict material downwelling beneath slabs^58^. However, recent geodynamic models indicate the possibility of sub-slab upwellings^59^, which could depress the 410. One possibility is that upward flow is facilitated through a hole in the slab at a depth of 400–500 km beneath Guadeloupe, which has been inferred on the basis of body-wave tomography^6,28^ and azimuth anisotropy^60^, perhaps aided by a complex folding slab geometry that requires diversion of mantle flow^6^. Similar flow has been interpreted from seismic constraints in Java^61^ and the northwest Pacific^62^. The greatest 410 depression at the north edge of the study area (with a maximum of approximately 30 km) could be caused by thermal effects on Wd reaction. The slab temperature is nearly 400–600 K colder than ambient mantle at depth about 410 km (Extended Data Fig. 6); an approximately 30–50-km uplift on the 410 is expected on the basis of a Clapeyron of 2.9 MPa K^−1^. Another factor can also affect the depth of the 410, for example, the Wd stability field is expected to shift to lower pressure by about 1 GPa (roughly 30 km)^27^ when nearly water saturated (that is, >0.5 wt%).

### Normal or uplifted 660

The lack of a 660 anomaly in the east and the slight uplift in the west (Fig. 2b) may be explained by a combination of factors. There is no expected upwelling or downwelling east of the slab, therefore the lack of a 660 anomaly, in turn, may indicate that the absolute depths are reasonable and there is little systematic offset owing to migration uncertainties. In the west, there may be some degree of upwelling, which may reflect a more complex flow pattern around the slab. Another possibility is that this could be caused by localized *V*_P_/*V*_S_ anomalies, for example, a decrease in *V*_P_/*V*_S_ of about 0.02–0.04 in the MTZ. Regardless, it is at the edge of our study area and we have no strong interpretation of the observation.

### Thermal and thermodynamic modelling

To test the expected phase boundary topography in the Antilles subducted slab, we ran a set of kinematic thermal models for a slab with constant dip and variable age and sinking velocity appropriate for the history of subduction at the Lesser Antilles arc (LAA). Slab and mantle temperatures are computed by solving the 3D energy equation including heat production in the upper plate and adiabatic heating throughout the domain, for an imposed velocity field. Slab velocities are assigned on the basis of the plate kinematics. Analytical solutions for a constant viscosity mantle are used for mantle flow above and below the slab while ensuring that the velocity field satisfies continuity^63^. For the cases considered here, we do not vary any properties along-strike and analyse a 2D vertical cross-section of the resulting thermal structure. Further details of the method, boundary conditions and thermal parameters used can be found in ref. ^63^. MTZ temperatures are controlled by the slab thermal parameter, that is, by incoming plate age and sinking velocity. We consider four different cases A to D that capture possible LAA subduction scenarios and predict a range of possible slab temperatures (represented in Extended Data Fig. 6 by minimum-temperature slab geotherms):

A. A reference case with a constant age at the trench of 80 Myr and a constant convergence velocity of 2 cm per year, consistent with the current age and velocity of subduction at the LAA. For the slab dip of 55°, on the basis of tomography (Fig. 3), this corresponds to a sinking velocity of 1.6 cm per year and the upper mantle part of the slab represents the past 40 Myr of subduction. This endmember case predicts the lowest slab temperatures (Extended Data Fig. 6).
B. Comparison of the history of plate motions and the distribution of slab fragments below the LAA^6^ indicates that the upper mantle below the LAA may contain material from the past 70–80 Myr of subduction. We therefore consider a second case with the same 80-Myr age at the trench as model A but with half the sinking velocity. This results in a warmer slab than model A.
C. From our reconstruction of plate age at the trench^6^ based on the plate model in ref. ^64^, we know that plate age at the trench varied notably as the Proto-Caribbean including its extinct spreading ridge were consumed. Below Dominica, which is approximately in the centre of our study region, some of the youngest material subducted, with plate ages at the trench changing from 40 Myr at 70 Ma, increasing to almost 60 Myr by 55 Ma, decreasing to 25 Myr at 38 Ma and then steadily increasing to the present-day age of 80 Myr. Convergence velocities also varied over this time, from velocities greater than 4 cm per year at 70 Ma to 1.5–2.0 cm per year today. We ran a case with this variation in age and velocity at the trench. In this case, the upper mantle part of the slab only corresponds to the past 23 Myr of subduction. Owing to the younger and variable ages, this results in a much warmer slab than models A and B and a more variable shape of the slab geotherm.
D. Finally, we consider a case such as C but with a third of the sinking velocity, which results in an upper mantle slab corresponding to most of the past 70 Myr of subduction. The lower sinking velocity makes the slab of this model the warmest of the four cases considered.

### Seismic modelling of required basalt percentages

To examine the effect of the basalt composition on the nature of the discontinuities near 660, we conducted receiver function synthesis across a range of 1D compositional models. We considered a range of mechanically mixed compositions from pure harzburgite (*f* = 0) to pure basalt (*f* = 1) between 600 km and 800 km depth and a pyrolitic mechanical mixture (*f* = 0.18) at all other depths. For comparison with the receiver functions, we considered the velocity profiles for the minimum slab temperature geotherms of our four thermal cases.

**Extended Data Fig. 9:**
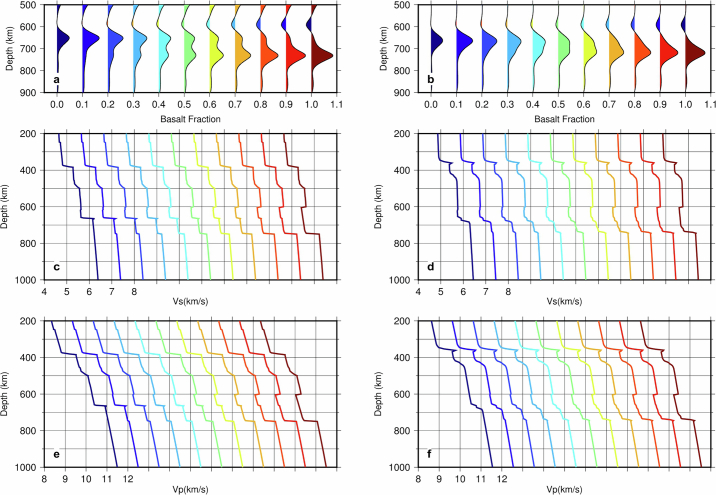
1D synthetic receiver functions and corresponding seismic velocities profiles. **a**, **c** and **e** based on the slab geotherm of model D (Dominica plate motion history and one-third of the slab-sinking velocity), which gives the warmest estimate of the geotherm. **b**, **d** and **f** based on the slab geotherm of model A (age = 80 Myr, velocity = 2 cm per year), which yields the coldest estimate of the geotherm.

To convert the slab thermal structures to seismic velocity structure, we used the thermodynamic modelling techniques described in ref. ^3^, using the code Perple_X (ref. ^65^) with the database for mantle minerals from ref. ^66^ and compositions from ref. ^67^. Synthetic seismic records were generated by a plane-wave propagator matrix algorithm^68^, which was subsequently used to compute synthetic receiver functions through the same time-domain integral deconvolution method applied to real data. Synthetic receiver functions based on the geotherm of the Dominica model are shown in Fig. 4b. We also show examples of models A and D, which correspond to the two endmembers of the predicted geotherms (Extended Data Fig. 9).

### 2D full seismic waveform modelling

To further investigate the robustness of our observed superdeep 660 phases and potential artefacts related the interaction of the wavefield with the slab, we calculated 2D full-waveform synthetic seismograms. We used the thermal structure from thermal model C as reference and considered a range of compositional models to generate seismic structures:

A. Mechanical mixture (MM). A reference case with constant composition of a pyrolitic mechanical mixture with a 0.18 basalt fraction, that is, no oceanic crust included (Extended Data Fig. 3a). This endmember case predicts the effects of the teleseismic wavefield interacting with the thermal structure of the subducting slab.
B. Layered basalt accumulation (LBA). In this case, we introduced a crust and a depleted mantle layer within both the slab and the upper plate. The crust, with a thickness of 7.5 km, was assumed to consist of pure basalt (*f* = 1.0). Below the crust, a depleted lithosphere layer, 20 km thick, consisted of pure harzburgite (*f* = 0.0). Within the depth range of 590–850 km, the composition of the slab was set to pure basalt (*f* = 1.0) across its full mechanical thickness (that is, overriding composition of crust, depleted lithosphere and part of the rest of the lithosphere). The background composition elsewhere consisted of mechanical mixtures with a fraction of *f* = 0.18 (Extended Data Fig. 3b). This endmember case lets us evaluate the impact of compositional heterogeneity within the slab on the nature of the near 660 discontinuities.
C. Layered basalt rectangle (LBR). This is similar to the LBA case but with a rectangular region of pure basalt. This rectangular region spanned depths from 590 to 850 km, with a horizontal extent of 200 km with the right-most corner at the position of the mechanical base of the slab at 590 km depth (Extended Data Fig. 3c). This endmember case lets us evaluate the influence of the horizontal scale of the basalt layer on the nature of the near 660 discontinuities.

**Extended Data Fig. 10:**
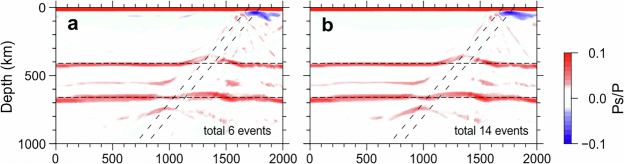
Migrated synthetic receiver function results. Migrated synthetic receiver function results for the LBA case using records from six synthetic seismic events, three angles from the left and three from the right (**a**), and 14 synthetic events, seven angles from the left and seven from the right (**b**). The Gt-transition-associated discontinuity, the positive phase below the 660-km discontinuity near the slab, is evident in both results. This suggests that, although the synthetic result with extra events presents a cleaner image, the result based on the six synthetic events remains robust enough for studying the behaviour of the 660-km discontinuity.

Synthetic seismic records were generated for receiver function analysis using the 2D spectral-element-method code^69,70^. Stations were assumed to be positioned at 5-km intervals on the surface. For each case, we considered plane waves incoming at angles of 40°, 55° and 70° from both the left and right directions. This resulted in a total of six incoming plane waves for each case. To compute the receiver functions, synthetic seismic records were processed using the same methodology applied to real seismic records—an iterative time-domain deconvolution method with a Gaussian width of 0.7 s. Subsequently, the synthetic receiver functions were depth-migrated on the basis of a 1D velocity model derived from the background of the 2D model (Extended Data Fig. 3g–i). We also conducted tests with a broader range of incoming plane waves, specifically at 5° intervals from 40° to 70° for both left and right directions. Although the synthetic migrated images with the extra incoming plane waves were cleaner, the result based on the six incoming plane waves proved to be sufficiently robust for the study of the 660 discontinuity behaviour (Extended Data Fig. 10).

In the MM case, the synthetic results reflect variations in MTZ structures in response to thermal effects, including an uplifted 410 discontinuity and a slightly depressed 660 in the vicinity of the subducting slab. The 660 discontinuity is slightly uplifted behind the slab, which is considered artificial, as the high-velocity slab is not taken into account during the migration process. No obvious phases are observed at depths greater than 700 km in this case.

In the LBA case, a superdeep 660 discontinuity appears at approximately 750 km depth near the subducted slab, representing the expected Gt phase transition. In the LBR case, the superdeep 660 discontinuity extends to the area behind the slab, at which there is prescribed enrichment in basalt. Meanwhile, the Rw-related discontinuity at about 660 km depth becomes undetectable in the basalt-enriched area, aligning well with our observations. The observed DNP is not prominent in the synthetic results and various factors may contribute to this outcome, as discussed in the 1D seismic modelling section.

## Online content

Any methods, additional references, Nature Portfolio reporting summaries, source data, extended data, supplementary information, acknowledgements, peer review information; details of author contributions and competing interests; and statements of data and code availability are available at 10.1038/s41586-025-08754-0.

## Source data

Source Data Fig. 2

Source Data Fig. 3

Source Data Extended Data Fig. 1

Source Data Extended Data Fig. 2

## Figures and Tables

**Extended Data Fig. 7:**
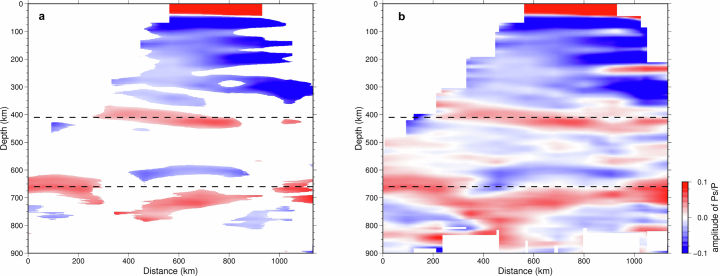
Vertical cross-sections through the migrated receiver functions along profile AA′ in Fig. 1. In panel **a**, grid cells with confidence level <95% are masked. Panel **b** shows all grid cells in which more than five receiver functions were stacked.

## Data Availability

Seismic data were downloaded through IRIS Web Services, including the following seismic networks (http://ds.iris.edu/mda): (1) the CU (Caribbean Network; Albuquerque, 2006; 10.7914/SN/CU); (2) the G (EOST, IPGP, 1982; 10.18715/GEOSCOPE.G); (3) the GL (IPGP, 2020; 10.18715/guadeloupe.gl); (4) the MQ (IPGP, 2020; 10.18715/martinique.mq); (5) the NA (KNMI, 2006; 10.21944/dffa7a3f-7e3a-3b33-a436-516a01b6af3f); (6) the TR (UWI, 1996; 10.22201/igeof.00167169p.1996.35.3.458); (7) the WI (GNSS, IPGP, 2008; 10.18715/antilles.WI); (8) the XZ (VoiLA Network; KIT, 2016; 10.7914/SN/XZ_2016). Source data are provided with this paper.

## Code availability

The method and codes used are standard and widely used and are detailed in Methods. Figures were made using Generic Mapping Tools and MATLAB.
